# The experience of daily life of acutely admitted frail elderly patients one week after discharge from the hospital

**DOI:** 10.3402/qhw.v10.27370

**Published:** 2015-06-01

**Authors:** Jane Andreasen, Hans Lund, Mette Aadahl, Erik E. Sørensen

**Affiliations:** 1 Department of Clinical Medicine, Aalborg University, Aalborg, Denmark; 2 Department of Physiotherapy and Occupational Therapy, Aalborg University Hospital, Aalborg, Denmark; 3 Research Unit for Musculoskeletal Function and Physiotherapy, Institute for Sports Science and Clinical Biomechanics, University of Southern Denmark, Odense M, Denmark; 4 Centre for Knowledge Based Practice, Bergen University College, Bergen, Norway; 5 Research Centre for Prevention and Health, The Capital Region of Denmark, Glostrup Hospital, Glostrup, Denmark; 6 Clinical Nursing Research Unit, Aalborg University Hospital, Aalborg, Denmark

**Keywords:** Older people, hospital admission, transition, interviews, qualitative, care

## Abstract

**Introduction:**

Frail elderly are at higher risk of negative outcomes such as disability, low quality of life, and hospital admissions. Furthermore, a peak in readmission of acutely admitted elderly patients is seen shortly after discharge. An investigation into the daily life experiences of the frail elderly shortly after discharge seems important to address these issues. The aim of this study was to explore how frail elderly patients experience daily life 1 week after discharge from an acute admission.

**Methods:**

The qualitative methodological approach was interpretive description. Data were gathered using individual interviews. The participants were frail elderly patients over 65 years of age, who were interviewed at their home 1 week after discharge from an acute admission to a medical ward.

**Results:**

Four main categories were identified: “The system,” “Keeping a social life,” “Being in everyday life,” and “Handling everyday life.” These categories affected the way the frail elderly experienced daily life and these elements resulted in a general feeling of well-being or non-well-being. The transition to home was experienced as unsafe and troublesome especially for the more frail participants, whereas the less frail experienced this less.

**Conclusion and discussion:**

Several elements and stressors were affecting the well-being of the participants in daily life 1 week after discharge. In particular, contact with the health care system created frustrations and worries, but also physical disability, loneliness, and inactivity were issues of concern. These elements should be addressed by health professionals in relation to the transition phase. Future interventions should incorporate a multidimensional and bio-psycho-social perspective when acutely admitted frail elderly are discharged. Stakeholders should evaluate present practice to seek to improve care across health care sectors.

This study focuses on acutely admitted frail elderly patients in transition from the hospital to home. The frail elderly in general are at higher risk of negative outcomes such as disability, dependency, low quality of life, hospital admissions and readmissions, need for long-term care, and death compared to non-frail elderly (Fried et al., 2001; Gobbens, Van Assen, Luijkx, & Schols, 2012; Hubbard, Andrew, Fallah, & Rockwood, 2010; Rockwood et al., 2005). Frail elderly are especially at risk for developing a hospitalization-associated disability between the onset of the illness and the discharge from the hospital (Covinsky, Pierluissi, & Johnston, 2011). Acutely admitted elderly patients are a large group where approximately 20% are at risk of readmission within 30 days (Sundhedsstyrelsen, 2009; Watkins, Hall, & Kring, 2012). Readmissions have large personal consequences for the patients and relatives, as well as economic consequences for society (Sundhedsstyrelsen, 2009).

When the term “frailty” was introduced in the mid-1970s; at first a biomechanical and physical definition of frailty dominated (Abellan Van Kan et al., 2008; Gobbens, Luijkx, Wijnen-Sponselee, & Schols, 2010a). Recently, a broader definition with a multidimensional (bio-psycho-social) integral perspective of frailty was introduced (Gobbens, Luijkx, Wijnen-Sponselee, & Schols, 2010b; Levers, Estabrooks, & Ross Kerr, 2006; Markle-Reid & Browne, 2003). Both perspectives of frailty, as a medical syndrome (Morley et al., 2013) and the integral approach (Gobbens et al., 2010b; Gobbens, Van Assen, Luijkx, Wijnen-Sponselee, & Schols, 2010), underline that frailty is a threatening condition for the elderly that should be identified by using validated and feasible screening tools. It is also underlined that frailty is a manageable condition where the aim is to prevent or delay adverse consequences (Gobbens et al., 2010b; Morley et al., 2013). Gobbens et al. (2010) defined frailty as “a dynamic state affecting an individual who experiences losses in one or more domains of human functioning (physical, psychological, social) that are caused by the influence of a range of variables and which increases the risk of adverse outcomes.” The integral definition, in addition to physical aspects, including psychological and social aspects and the relationships between them (Gobbens et al., 2010b), reflect the position of the authors of this article. This definition provided by Gobbens et al. is the theoretical perspective used as a starting point for understanding the concept of frailty.

Community-dwelling frail elderly described a lower quality of life than the non-frail and it seemed that quality of life decreased as frailty increased. The frail elderly reported social contact as the most important aspect of quality of life whereas the non-frail reported health (Puts et al., 2007). Nicholson, Meyer, Flatley, Holman, and Lowton (2013) described how the frail elderly experienced loss of autonomy and functional capacity, but they also reported creativity in handling the shortcomings from a life with frailty. Other studies exploring the experience of daily life of community-dwelling frail elderly showed that their experiences varied, but at the same time it seemed that the frail elderly in general experience different kinds of struggles in relation to management of daily life (Haak, Malmgren Fange, Iwarsson, & Dahlin-Ivanoff, 2011; Katz, Holland, & Peace, 2013; Tollén, Fredriksson, & Kamwendo, 2008; Van Campen, 2011; Ebrahimi, Wilhelmson, Eklund, Moore & Jakobsson, 2013; Ebrahimi, Wilhelmson, Moore & Jakobsson, 2012; Nicholson, Meyer, Flatley, Holman & Lowton, 2012).

A systematic literature search identified a total of two studies that described experiences of frail elderly in relation to hospital admission and discharge (Åberg, Sidenvall, Hepworth, O'Reilly, & Lithell, 2005; Kristensson, Hallberg, & Ekwall, 2010). Fifteen acutely admitted frail elderly patients were interviewed about life satisfaction, 1 and 6 months after discharge. Activity, independence, and adaptation were important elements for life satisfaction. Adaption was defined as being the means to cope with negative consequences for life satisfaction caused by activity limitations and decreasing independence (Åberg et al., 2005). Kristensson et al. (2010) found that frail elderly experienced a sense of having power or being powerless in relation to encounters with staff and their satisfaction with the care and services provided. In summary, varied experiences related to being frail and old were seen and the literature search revealed that many frail elderly faced challenges and defeats that were beyond their own control. No studies focused on the first few days after discharge of frail elderly patients even though this is a high-risk period; a peak in readmissions is seen in that time span and frail elderly medical patients have an increased risk of hospitalization-associated disability after discharge (Covinsky et al., 2011; Misky, Wald, & Coleman, 2010; Sundhedsstyrelsen, 2009). Knowledge about the experience of daily lives of the frail elderly may be crucial to identify and address issues related to the transition from hospital to home.

The aim of this study was to explore how the frail elderly experience daily life 1 week after discharge from an acute admission to the hospital.

## Methods

### Methodological approach

The methodological approach was interpretive description (ID) described by Thorne (2008) and Thorne and colleagues (Thorne, Kirkham, & MacDonald-Emes, 1997; Thorne, Kirkham, & O'Flynn-Magee, 2004). The foundation of ID is smaller-scale qualitative investigations of clinical phenomenon for the purpose of capturing themes and patterns within subjective perceptions, thereby generating an ID capable of informing clinical practice around complex clinical questions (Thorne, 2008; Thorne et al., 2004). The reporting of this study is in accordance with the Consolidated Criteria for Reporting Qualitative Research (COREQ) (Tong, Sainsbury, & Craig, 2007).

### Study setting

The setting was the Danish health care sector. Health care services in both the primary (communities) and the secondary sector (public urban hospitals) are free of charge and independent of the individual's income and insurance. In the primary sector inspectors evaluate and define the need and amount of health care services.

### Study participants

The participants were acutely admitted frail elderly patients over 65 years old. Patients with terminal illness and severe dementia were excluded. A purposive sampling was used and participants from seven medical wards at a university hospital were recruited. A wide range of diagnoses and degrees of frailty and demographic characteristics were secured to catch nuances and to facilitate maximum information and sufficient data richness (Thorne, 2008). Shortly before discharge 22 patients in total were informed about the study and invited to participate. In all, 20 patients gave consent, but two died before discharge; one became ill and discharge was delayed; and three withdrew consent: one due to lack of resources, one due to distress and anxiety, and one because she did not want to be audiotaped. The researcher contacted the patients by telephone 3–5 days after discharge and repeated the information and the aim of the study. If the participant still consented, the interview took place in the participant's home approximately 1 week from discharge. Fourteen participants: seven men and seven women, gave informed consent. The participants filled in the Danish version of the Tilburg Frailty Indicator, which is a self-reporting screening tool for frailty with a bio-psycho-social approach (Andreasen, Sorensen, Gobbens, Lund, & Aadahl, 2014; Gobbens et al., 2010). The participants were all frail, some very frail, according to the Tilburg Frailty Indicator. Mean age of the 14 participants was 80.6 years (range 69–93 years) and all had comorbidity. Three of the participants wanted their partner to be present, and the partners contributed during parts of the interview. The interviews were carried out from November 2013 to September 2014. Characteristics of the participants are presented in Table I.

**Table I:** Participant characteristics.

Participant pseudonym	Age/sex	Marital status	Educational level	Primary diagnosis	Urban/rural residence	Length of stay/days	TFI* total score	TFI physical score	TFI psychological score	TFI social score
Anders	79/M	Partner	Elementary	Pneumonia	Rural	12	12	8	3	1
Maria	78/F	Partner	Elementary	Emboli	Urban	12	5	4	1	0
Peter	71/M	Single	University/college	Bilat. amputee	Urban	24	7	5	1	1
Leonora	93/F	Single	Elementary	Fall	Urban	5	7	5	1	1
Allan	87/M	Partner	Elementary	Pneumonia	Urban	6	7	6	0	1
Richard	82/M	Single	Elementary	Brain abscess	Urban	73	10	5	3	2
Anna	72/F	Single	Elementary	Weight loss	Urban	13	9	7	1	1
Anne	87/F	Single	University/college	Hypoglycemia	Urban	9	11	7	2	2
Elisabeth	81/F	Single	Elementary	Renal failure	Rural	28	7	2	3	2
Simon	69/M	Partner	Elementary	Bilat. pneumonia	Urban	4	7	4	2	1
Johannes	83/M	Partner	Elementary	Pancreatitis	Rural	16	6	6	0	0
Edith	83/F	Single	University/college	Type 2 diabetes	Rural	7	9	5	2	2
Mick	89/M	Single	Elementary	Pneumonia	Urban	9	9	6	1	2
Betty	75/F	Single	Elementary	Dizziness	Rural	15	8	4	2	2

A total cut-off score of five on the Tilburg Frailty Indicator is defined as frail, with a possible maximum score of 15 and a minimum score of 0.

### Data collection

Data were gathered using interviews (Kvale & Brinkmann, 2009). The role as interviewer was an encouraging, non-normative neutral facilitator so that the participants could explain themselves as fully as possible (Thorne, 2008). A semi-structured interview guide was pilot-tested on two frail elderly persons. The first and last author discussed the content and process and concluded that no changes should be made (Table II). The interviews lasted from 29 to 80 min. All interviews were audiotaped and transcribed verbatim by the first author immediately after as the analysis is an ongoing process starting immediately after data collection (Thorne, 2008). The transcriptions resulted in a total of 185 pages (text type Calibri, size 11).

**Table II:** Main topics of the interview guide.

1. How do you feel at the moment?
2. Can you describe a typical day and tell me what you experience through a typical day?
3. What is a good daily life to you in a broad sense, and how do you experience your own daily life at the moment?
4. What is important for you in relation to daily life?
5. The way things are in your life right now, how do you experience and how do you manage the circumstances?
6. Are there any elements or issues in relation to the transition from hospital to home you want to tell me about?
7. Is there anything else you want to tell me, something you find important?

### Ethics

The Ethics Committee in the Region of North Jutland, Denmark, stated that no approval was necessary because this kind of study by law does not need approval (www.cvk.sum.dk). The study was approved by the Danish Data Protection Agency (number 2008-58-0028). All patients were given written and oral information about the study and informed written consent was obtained from all participants prior to participation. Confidentiality and anonymity were secured and participants were told that their statements would be treated in confidence and that all quotations would be anonymous. As the participants were frail and newly discharged from the hospital, the interviewer was careful in securing the integrity of the participants. It was emphasized that the participants could withdraw consent at any time without consequences for present or future treatment.

### Data analysis

The analysis was a dynamic process even though a step-by-step approach was used, as the authors went back and forth in the analytic process to question, define, decide, and conclude as recommended (Thorne, 2008). Initial readings of the interviews were done continuously to get a sense of the full material. The first analytic interpretation was open to ensure no “early disclosure” of data (Thorne, 2008). Preliminary categories were enlightened and extracted from the data material by using a constant comparative approach from parts to the whole and vice versa (Thorne, 2008; Thorne et al., 2004). An iterative approach was conducted to qualify and clarify the preliminary categories resulting in four main categories.

## Results

The four main categories named “The system,” “Keeping a social life,” “Being in everyday life,” and “Handling everyday life,” showed that the frail elderly, in some cases, found similar aspects important. In other cases they differed, meaning that elements that affected and frustrated some participants were not an issue for others. The four categories are described in the following section. Quotations are used to present and describe the findings. All names used are pseudonyms due to anonymity.

### The system

“The system” understood as the health care sector in a broad sense greatly influenced the participants’ experience of daily life in relation to health care service, transition, and medicine. The provision of health care and/or social services (cleaning) was a matter of great concern and frustration or it was satisfactory and uncomplicated. The latter was especially the case if the same health care worker provided service and saw the client as a human being and fulfilled her/his needs. Leonora who had lived alone in her apartment for several years appreciated a close relationship to her care worker in this way: “I have had the same girl [care worker] for many years and she knows when my bedding needs to be changed and all things like that, and she is very, very sweet and I am happy about that.”

Richard who had been in the hospital for a long time could not figure out why all the different people visited him and felt it was needless. He felt it was disturbing his effort to get back to his daily routines after discharge; the system oversupplied. As he said: “Somebody is coming all the time to help with this or that. Actually I asked for help in the mornings and evenings myself, but it is actually needless. I am already up and washed and all before they come.”

The system either undersupplied or supplied but not in relation to expressed needs. In situations where needs were not met after discharge the patients felt treated as objects and like insistent and tiresome cases, and the feeling of being objectivized had negative consequences. Anne was frustrated as the things she needed help to get done were not possible, only a standard cleaning package of vacuuming and floor washing was provided. Anne was asked if she needed more help, and she answered: “No actually not, as they are not allowed to do what I want them to do, for instance cleaning the refrigerator, cleaning windows, amongst other things.” Anders and his wife were living on a farm in the countryside. They were offended by the small amount of help they could receive although their situation was complex and demanding. They felt it was a battle and felt violated. Anders had apoplexy and a bilateral pneumonia and was still weak after discharge. His ambulation skills were very much affected and it was a physical effort to assist him. He got up at seven o'clock to have coffee and breakfast with the help of his wife. Afterwards he was assisted back to bed again and waited for home care. Anders and his wife felt left alone and let down by the system to which they had been paying taxes their entire lifetime. The disillusioned and tired wife expressed her feelings:

I am just so disappointed with the municipality; there is actually no help from them, it is rotten, it is all calculations about money. There are no humans in it at all. It sounded uhh so fine, but coming here telling us that they have 14 min, that is what they are saying when they arrive. I find it too bad. The time varies between 9 and 20 to 11 and we do not know when they arrive.

Participants experienced a transition to home that felt insecure and in some instances unsafe and even dangerous. They experienced that you cannot trust the system as information from the hospital about appointments made with the health professionals in the municipality did not take place as expected. This led to a feeling of a system that really did not take proper and professional care of you as an ill human being in need. Peter, who had both legs amputated, came home in the late afternoon in a wheelchair after 3 weeks in the hospital. He was asked if he had any considerations in relation to the transition, and he answered:

Ohh will you stop it! I came home to a house, where nothing was taken care of. Nothing! There was a “bedpan chair” with no bedpan beneath, so I could just shit on the floor. That was it! So everybody [staff at hospital] says, we are ready now, transportation is ordered and so forth and then you return to a house where you find a toilet chair, where there is nothing to pick up in. Thank God I had a bricklayer bucket in the utility room, otherwise only the floor would have been left. Something like that is not professional. I came home Thursday and then Annette [the home carer] came Friday starting to phone God and everybody, and she just got this message everywhere: “But this is Friday, so it can′t be until next week.”

The feeling of being let down by the system and dependent upon the help and care from neighbors after discharge and the feeling of being discharged without a diagnosis or explanation produced a feeling of anxiety and uncertainty. As it was said by Betty:

But you know, I live alone right. And what if it [the illness] came back? Now when they haven't figured something out. I was afraid of that. And I found it awful that is for sure. And did I not have such good neighbors; I would never have succeeded, never.

Richard, on the other hand, did not feel that discharge was too early when asked about the transition: “No it was a nice and soft transition, so to speak; it was not a violent transition.” An experience of a well-prepared and timely discharge resulted in a harmonic feeling and readiness for coming back to daily life at home.

Medicine was an issue too. Some got more than 10 different types of medicine and one got 18. Lack of coordination of medicine between the physician in private practice and the public urban hospital caused frustrations. The participants had a hard time getting an overview of their medicine, as the name of the preparations changed all the time and also the types of medicine changed. Different approaches to the prescribed medicine were used. New medicine could be met with full control and was expressed with satisfaction, just like Edith, who was newly diagnosed with diabetes mellitus, said: “Yes, I think I am doing quite well. Well that is the way it is now and there is nothing else to do than dig in, you see?”

Another approach was to have confidence in the health professionals. Betty was asked if she knew what kinds of medicine she took. Her answer was: “No, that is for the nurse. I do not really use my head for that at all.” Finally, one did not at all feel safe and secure in handling the medicine but was confused and expressed a high level of anxiety. Elisabeth expressed worry and anxiety and was scared of taking the wrong medicine in this way:

These pills, I think a lot about them because I am not at all used to them. And I am always scared that a mistake should happen. What if I get something I cannot tolerate, that is what I am the most scared of?

### Keeping a social life

“Keeping a social life” described the participants’ experiences of relationships and loss, and their efforts in keeping social relations despite physical or psychological challenges in daily life. Positive and strong relations with a spouse, family (especially children), friends, and/or neighbors were emphasized as maybe the most important in daily life. The social dimension characterized by a feeling of closeness and understanding was emphasized. Becoming ill and dependent of one's spouse, children, or neighbors could on the other hand be stressful, and a feeling of being a burden in relation to the closest relatives caused anxiety and guilt. A loss in social life in which somebody close has died was frequently described and this created consequences, such as loneliness and no one to exist for, and this seriously negatively affected the returning to home. As Edith stated: “Yes, contact with my children is important for me. I don't think anything else means that much anymore. I am very much a family person. It means the world to me.”

Although very important, it was also an element that was troublesome because relationships had changed due to illness and thereby the relationship with the spouse and children changed. Anders was asked what was really important in daily life and he said:

What is really important for me is, in fact, that our marriage is functioning and I think it still does, but I have, in fact, been nervous that it maybe would break down, because this has been too big a strain.

Mick, an 89-year-old man who lost his wife 2 years ago reflected: “I was definitely happier with life when I had her around and then I had something to work for and exist for and something that made the days go by.”

Another type of loss was loss of contact with children or friends due to illness, abuse, or problematic relationships. Problematic relationships with children or friends show how conflicts caused stressful thoughts and resulted in a distressed social life that was compromised and reduced. Betty's grief concerned one of three sons, whom she had not seen for 6 years:

We were down there [at a restaurant to celebrate Betty's 70th birthday] to eat and then they left in the night and drove to Northern Sweden and I have not heard from them or seen them since then. That is hard.

### Being in everyday life

“Being in everyday life” described the influence of mood and mindset on daily life. Some participants experienced daily life as a struggle, whereas others experienced it as more peaceful and satisfying. The way of handling challenges depended on mood and view on life. However it also seemed that the more challenges the elderly experienced the more difficult it was to keep a positive mood. Psychological as well as social and physical resources influenced the way the patients acted and coped after discharge: the more resources, the more there seemed to be a tendency to keep up spirit and initiative. Maria was living in a house in the city with her husband and she was very tired after discharge, otherwise she did not experience physical disability. They received no help from the community. Maria defined good health as: “Yes, but that is being able to get up in the morning, and you are happy and satisfied, and, well, I think I have been that all my life.” Leonora has been living alone in her apartment on the third floor for three decades. In the late afternoon she was sitting quietly in the dark. She suffered from severe back pain, had no appetite, and felt weak. She was asked what she valued the most in her life and replied:

Nothing is important anymore. No, there is no meaning to anything. Sometimes I want to die, because there is no meaning to anything. The kids have grown old and the grandchildren and all that, so I have experienced that.

Mick, whose wife had passed away recently, could not do what he used to and he felt odd and down:

I cannot tell you how it is; it is strange; it is kind of tingling all over. It is like I am lacking a little pep. I do get “the happy pill,” but maybe I need double of it, I don't know. It is a bit difficult to say. One is sent home from the hospital to an empty apartment, and one of the kids is always here then, but they leave too you know. Do you know what I think? I have always been happy in life and always been involved and then suddenly you can't anymore, but I am still happy in life, love to live. But today I feel like I say; I wish I could close my eyes; that is the way I feel today.

It seems like being inactive, not participating in meaningful activities, and/or feeling alone shortly after discharge caused distress and a wish to die may emerge. Mindset revealed the different ways participants dealt with their situations. Leonora had kind of resigned to the situation: “I am as handicapped as one can be, but it is of no use just to sit down and say: ‘What am I going to do?’ I just have to take the day as it comes, right?” Maria on the other hand was convinced that your own attitude was important:

Don't you think that it depends on how you yourself are minded. You know if you give up on it all and think never mind. I think you need to have an attitude saying “Now I am heading forward and I myself have to take care.”

Betty, who lost her husband 6 years ago, found it important not to feel sorry for oneself and told her friends who said that time felt long: “No it is definitely not; you make it that yourselves, because you feel sorry for yourselves; don't feel that.”

### Handling everyday life

“Handling everyday life” was influenced by physical constraints and symptoms and was closely related to participants’ present illness or to a general decrease in physical functioning. The focus on health and health-related symptoms was stressful for the elderly and for some it clearly created restlessness and worries about getting more ill. Spending time participating in activities was important but not all were capable of doing the activities they valued anymore, primarily due to their physical condition. Being tired was a very common condition after discharge and created a vicious circle as tiredness resulted in inactivity. Even though the participants recognized this, they could not mobilize the energy or will to break the circle at this particular time. The feeling of being inactive in daily life, for instance, due to tiredness or incapability, was unsatisfactory. Watching television became a replacement. Peter was concerned about his future mobility in this way:

So I am anxious about whether I will be locked up in this house or I may happily be able to use prostheses, and for God's sake please do not lock me up in here, but give me a handicap vehicle, so that I can get around.

The absence of pain was a major relief, or it was symptoms related to the lungs such as the color of mucus or breathing that were important. Simon had chronic obstructive pulmonary disease and was treated with permanent oxygen: “If I get the humidity from the water [when showering] and all that, I shall, I just can't, then it shots down the [he points at his lungs]. I am beside myself, when I am out there!”

Participants emphasized the possibility of exercising as important. Allan was waiting for the municipality to offer exercise, but he did not agree on the way the offer was handled. The offer was short-term and therefore a regain of mobility and strength would diminish shortly after:

I only have one goal and that is to get going again, to get a possibility for rehabilitation and self-training afterwards. You get 10 sessions of training, and when the 10 sessions are over it is goodbye. They could as well have spared the offer, because you will have the same result afterwards.

However, overall, participants did not explore knowledge or awareness of their conditions and illnesses and how they could improve their physical condition. A commonly experienced physical condition was tiredness, although participants had difficulty explaining how tiredness felt. Anders was talking about the day and said: “But it is actually spent mostly in bed; I am extremely tired all the time. Although I don't do anything, I am very tired.” It was difficult for participants to distinguish between physical and psychological tiredness. Some felt indolent and had to force themselves to do something. Hans, a former farmer of few words, said it like this: “Yes, indolence. Kind of indolence. Then one remains sitting.”

Participants emphasized the ability to manage daily activities such as cooking, reading, or knitting. Mick played petanque before but did not have the energy after discharge. When asked if he went to training or had help in daily life, he answered:

No, I walk around here on my own, and then I have two things I am grappling with. I do want to cook myself and then when I want to make soup, then some good bones or some chicken or something is needed, and then cutting and peeling potatoes and carrots and all that. And then a couple of hours have passed by, or three. And before I have finished the soup 3 hours have passed, and then I feel good.

Leonora on the other hand felt that not much was possible compared to earlier:

Oh I did so much: I sewed, I went to different centers and learned so many things, well yes, I have been active. I also went swimming and I would have liked to continue that, but I couldn't due to the long distance to the swimming pool, so I can't walk down there.

Anna lived alone in a small wooden house on the outskirts of town. She did not want much to happen during the day: “No I think they are one and the same. One day follows the other and I actually think that is very fine. I am not much into all those changes that are coming round and about.”

Without exception television was an important and time-consuming activity in daily life, some were selective about programs, others were not picky and watched television for many hours as this made the time go by. Leonora was asked what time she went to bed: “Oh it is sometimes at five or six o'clock; it depends on how I feel. But I have a television by the bed, so I can watch that. Then the time goes alright there.”

## Discussion

The results describe four main categories of importance in daily life: “The system,” “Keeping a social life,” “Being in everyday life,” and “Handling everyday life.” The four categories greatly affected the way frail elderly individuals experienced daily life. Different elements affected and were important in the life of the frail elderly, and these elements resulted in a general feeling of well-being or non-well-being. Whether participants felt well-being by feeling calm and in control or non-well-being by feeling restless and without control depended on how their needs were met and how they experienced their lives and challenges. There was a tendency that the frailer the participants were, the more they experienced stressors and struggles in daily life. Some felt calm and in control in daily life and expressed general well-being, whereas others experienced no control and restlessness, such as Elisabeth who was constantly worried about taking the wrong medicine, and Anders who felt restless and anxious all day. In relation to hospitalization and discharge Kristensson et al. (2010) focused on experiences in relation to the health care system 3 and 26 weeks after discharge. Åberg et al. (2005) investigated what older adults from a geriatric ward judged as important to be able to achieve life satisfaction in relation to rehabilitation. Our study, however, focused on the overall experiences of daily life 1 week after discharge and showed that some elements and stressors not only affect the physiological system, but also the psychological and social system. This study generates additional new findings, which are important to take into consideration in relation to the discharge situation. In particular the contact and communication with the health care system created frustrations and worries, but also physical disability, loneliness, and inactivity were issues of concern. Frail elderly has previously been described as a complex physiological system, where stressors disturb the homeostasis of the system, thereby increasing the risk of adverse consequences (Clegg, Young, Iliffe, Rikkert, & Rockwood, 2013; Fried, Ferrucci, Darer, Williamson, & Anderson, 2004; Nowak & Hubbard, 2009). These stressors may become detectable by looking at clinical, functional, behavioral, and biological markers (Fried et al., 2004). As a consequence of the aforementioned findings, health professionals should include psychological and social stressors as well, as this approach may contribute to better identification of stressors and thereby better understanding of the needs of the elderly. A bio-psycho-social approach would in addition have the profound consequence that involvement and perspectives of the frail elderly should be heard in decision-making related to transition and discharge. A study by Foss and Hofoss (2011) showed that this was still not the case for a large number of elderly hospitalized patients.

In our study, system failure was related to physical challenges, psychological or social elements such as loneliness, and loss or lack of support. Anders’ wife was the element that kept the system together and she was at risk of giving up, which would have huge consequences for Anders as well as for the municipality. The only alternative would be a nursing home. A system failure cannot be easily restored and is a complex and expensive task (Cook, 1998). It is therefore important to focus on homeostasis and an understanding of the system's complexity to avoid failure and ensure the well-being of the frail elderly. The complex system of the frail elderly individual is part of another complex system, the health care system, which consists of two health care sectors: a primary (communities), and a secondary (public urban hospitals) sector. It may therefore be understandable that the health care system does not succeed in all transition phases due to the complexity involved. However, in our study several stressors were identified shortly after discharge, such as the amount and type of help, help devices, and medication, all factors that can lead to system failure and to unnecessary suffering for the elderly (Rytter et al., 2010; Svanström, Sundler, Berglund, & Westin, 2013). The two health care sectors had difficulties coordinating, communicating, and coping with these issues. System failures can lead to adverse events like early readmissions, drug events, and falls (Laugaland, Aase, & Barach, 2012; Rytter et al., 2010; Sundhedsstyrelsen, 2009). A review from Laugaland et al. (2012) strongly indicated that targeted interventions were needed to improve transitions across health care settings. The health care system seems to need a defined strategy that captures the nature of complex systems so it becomes capable of identifying, adapting, and acting against stressors and potential failure in and between both sectors (Gray, 2014a, 2014b). The organizational structure may need to be redefined and reorganized to be capable of dealing with the complexity and secure the well-being of the acutely admitted frail elderly in relation to discharge.

The findings from this study contribute to important knowledge about the experiences right after discharge and these should be taken into consideration when elderly are discharged after an acute admission. Further research is needed to investigate how the discharge phase may be improved and especially to investigate the impact on the well-being of the elderly.

The 20 participants that were included in this study were extremely vulnerable and the majority had high frailty scores. The fact that two included patients died before discharge, two withdrew consent due to lack of resources and anxiety, and two of the interviewed participants were readmitted within the first week emphasizes the vulnerable state of the participants. The 14 interviews revealed positive elements but also plenty of potential stressors and unmet needs, all elements that greatly affected the experience of daily life. In particular, the experiences in relation to the transition to home were of importance for the participants, for instance, the character and amount of help, medication worries, communications gaps, and being alone and ill. Our study supports earlier research showing unsafe transitions in relation to elderly patients’ discharge from hospital (Hesselink, Schoonhoven, Plas, Wollersheim, & Vernooij-Dassen, 2013; Storm, Siemsen, Laugaland, Dyrstad, & Aase, 2014). The quality of the discharge for elderly patients aged over 75 years with multifaceted care needs, amongst other things, was impaired by lack of systematic information exchange between health care professionals and next of kin (Hvalvik & Reierson 2015; Storm et al., 2014). Furthermore, limited involvement and preparation for patients’ transition and the lack of a professional system and role competence of health care professionals were described. Hesselink et al. (2013) reported similar findings, although their study population was younger and healthier. This study adds in-depth knowledge of how significantly these issues, and not simply the quality of the discharge in terms of quality assessment indicators, affect the well-being of the frail elderly. A focus on the identification of these problems seems to be an important step in the process of improving transition, to secure well-being, and to avoid serious adverse events.

The majority of the participants did not mention the character of their illness and condition and it seemed as if they were unaware or did not pay much attention to the consequences of physical inactivity in relation to daily life. Frailty can be reversed as well as deteriorate and, in particular, exercising, nutritional support, and reduction of polypharmacy are evidence-based interventions (Morley et al., 2013). Today the health care system in Denmark provides cleaning help and personal help immediately after discharge. From an individual and a health economy perspective, in the future, practices such as physical training and dietary advice should be provided immediately after discharge, and also on a long-term basis.

### Methodological considerations

The participants met the predefined criteria of diversity in diagnosis, age, sex, and degree of frailty. Due to the sample size the findings may be dismissed as unique with no scientific value. However, there are aspects of the universal in the unique (Kvale & Brinkmann, 2009) and the study did provide rich and varied data to a degree that ensured a qualified and credible answer to the aim of this study.

A methodological consideration has been whether a week after discharge was too short a time span for the frail elderly to settle into the home environment, but adverse events more often occur shortly after discharge and in phases of transition (Hesselink et al., 2013; Storm et al., 2014; Sundhedsstyrelsen, 2009).

The first author primarily carried out the analytic process and was supervised by the last author who read and coded five interviews to secure agreement of the analytic process. All authors systematically followed the analytic process questioning and discussing the findings. This rigorous and transparent process was performed and presuppositions were continuously reflected upon, seeking to achieve trustworthy and credible findings (Thorne, 2008).

### Implications for practice

Health care professionals should inform the frail elderly in transition more thoroughly about their situation and of the consequences of their condition. Actively trying to decrease the level of frailty and knowing the consequences of not acting should be a necessity for frail elderly patients in relation to discharge. Consequently the frail elderly patients and their relatives should be more involved in decision-making and goal-setting in relation to discharge. The health care system takes special care of issues related to physical conditions, but the psychological and social issues reflected as restlessness, loneliness, or disillusion were also stressors that need to be addressed in future strategies, for instance, by the systematic involvement of social workers or volunteers.

Discharge of acutely admitted frail elderly patients should be based on mutual understanding of frailty as a complex bio-psycho-social system. A valid screening tool with a multidimensional approach may be an important element in systemizing the health care process within and between the two sectors in the future. Identification of the frail elderly at risk while hospitalized could make it possible to initiate a targeted intervention to avoid adverse events. More research is needed to address these specific challenges.

## Conclusion

This study provides in-depth knowledge on how acutely admitted frail elderly experience life 1 week after discharge. The patients’ experience of daily life 1 week after discharge from the hospital was affected by different factors. Contact with the health care system, social relations, mood, and constraints in handling daily life significantly influenced overall well-being or non-well-being and some of the elderly expressed great concern and worry in relation to their daily life. Therefore future interventions should incorporate a multidimensional perspective when acutely admitted frail elderly are discharged from the hospital. Stakeholders should evaluate present practice and maybe even consider a redefinition and reorganization of the health care system to ensure high quality of integrated care across the primary and secondary sector.
